# Development of a 3D-printed neonatal congenital diaphragmatic hernia model and standardisation of intra-operative measurement

**DOI:** 10.1007/s00383-023-05600-0

**Published:** 2023-12-26

**Authors:** Paediatric Surgical Trainees Research Network (PSTRN), George S. Bethell, Mary Patrice Eastwood, Jonathan J. Neville, Rachel Harwood, Sajeed Ali, Setthasorn Zhi Yang Ooi, Joshua Brown, Lucinda Tullie, Sesi Hotonu, Timothy J. Bradnock, Nigel J. Hall, SurgeryLabs PSTRN, Sofia Chacon, Reza Haghighi Osgouei, Jonathan J. Neville

**Affiliations:** University Surgery Unit, University of Southampton, Southampton, UK

**Keywords:** Congenital diaphragmatic hernia, Simulation, 3D-printed model, Thoracoscopic

## Abstract

**Introduction:**

Three-dimensional (3D) printing is frequently used for surgical simulation and training, however, no widely available model exists for neonatal congenital diaphragmatic hernia (CDH). The aim of this study was to develop a 3D-printed model of CDH and test interobserver variability in the simulated model for obtaining measurements of the diaphragmatic defect and ipsilateral diaphragm.

**Methods:**

A term fetal MRI (3.5 kg) of thorax, diaphragm and defect (15 mm × 5 mm) were delineated and segmented after parental consent to produce 3D-printed models. Consultant and trainee paediatric surgeons were invited to measure the posterior-lateral diaphragmatic defect and ipsilateral diaphragm. Mean measurement error was calculated (millimetres). Data are presented as median (range) and number/total (%).

**Results:**

An abdominal and thoracoscopic model were produced and tested by 52 participants (20 consultants and 32 trainees). Diaphragmatic defect via laparotomy measured 15 (10–20) mm (AP) × 16 (10–25) mm (ML) and thoracoscopically 14 (11–19) mm (AP) × 15 (11–22) mm (ML). Mean error per measurement was 4 (1–17) mm via laparotomy vs. 3 (0.5–9.5) mm thoracoscopically. Mean error was similar between consultants and trainees via laparotomy (4.3 vs. 3.9 mm, *p* = 0.70) and thoracoscopically (3 vs. 3 mm, *p* = 0.79). Error did not correlate with experience as operating surgeon via laparotomy (*β* = 13.0 [95% CI − 55.9 to 82.0], *p* = 0.71) or thoracoscopically (*β* = 1.4[95% CI − 6.4 to 9.2], *p* = 0.73.

**Conclusions:**

We have designed and built simulation models for CDH repair via laparotomy and thoracoscopically. Operators can reliably measure the diaphragmatic defect and ipsilateral diaphragm, regardless of surgical experience and operative approach.

## Introduction

Congenital diaphragmatic hernia (CDH) is seen in around 1 in 5900 live births [1]. Surgical repair is required in early life, once physiological stability is achieved. In survivors, a commonly seen complication following surgical repair is recurrence of CDH. This is more commonly seen following patch repair (27%), although it can occur following primary closure [2]. This discrepancy is thought to be related to defect size, as patch use tends to be for defects not amenable to primary closure. However, some surgeons advocate liberal use of patches, achieving low early recurrence rates with a tension free closure in smaller defects [3, 4]. To aid standardised reporting, classification of diaphragmatic defects was established by the CDH Study Group in 2008 [5]. However, post-mortem studies would suggest this underestimates the complexity of CDH [6].

Defect size has been shown to be an independent predictor of CDH recurrence, however, other operative interventions such as suture type, patch size, shape and type may also have an influence on long-term outcomes [7]. A multicentre, international prospective study looking at diaphragmatic repair in neonates is ongoing to understand defect phenotype and document repair technique. Accurate statistical analysis, interpretation by clinicians and implementation of findings into clinical practice, however, relies on the ability to accurately measure and report the diaphragmatic defect by surgeons. This can be challenging in neonates, due to size of the operative domain, associated morbidities, herniating viscera and the lack of an established measurement technique.

Surgical simulation has many forms and countless utilities. Three-dimensional (3D) printed models are used to simulate whole or parts of paediatric surgical procedures. These include pyloromyotomy, congenital heart disease and oesophageal atresia repair [8]. To our knowledge, there have been no previous reports of a 3D-printed model of CDH.

The aim of this study was to develop a 3D-printed model of CDH and test interobserver variability in the simulated model for obtaining thoracoscopic and abdominal measurements of the diaphragmatic defect and ipsilateral diaphragm.

## Methods

### Three-dimensional model development

Two models, with identical diaphragmatic defects, were designed and built to simulate the operative steps of CDH repair via laparotomy and thoracoscopy with assessment of interobserver reliability of measuring the defect. Parental informed consent was obtained to use a pre-existing MRI scan from a foetus with a prenatal diagnosis of CDH for the purposes of designing a surgical simulation model. Stereolithography (STL) files were created from the patient MRI using the open-source software ITK-SNAP (v.3.8.0, www.itksnap.org). The size and relative proportions of the thorax, diaphragm and defect were delineated and segmented. The computer-assisted design (CAD) software Blender 3D (Blender Foundation, USA) and Fusion 360 (Autodesk Inc, USA) were used to clean, refine, and modify the ribcage, spine and pelvis.

For the thoracoscopic repair model, the left hemithorax was isolated and orientated in a right lateral position to simulate access to the left hemi-diaphragm. The ribs were reinforced at the vertebral bodies and costochondral joints with geometric shapes, and the scapula applied to the posterior ribcage. Geometric shapes were also added to the sternal side of the ribcage to allow fixation to the model base. For the model simulating repair via laparotomy, the entire rib cage was isolated and combined with the spine and pelvis as one component. This component was reinforced with geometric shapes to add strength and allow fixation to the base, simulating a baby lying supine.

The ribcage was 3D-printed (Prusa MK3) using a flexible thermoplastic polyurethane (TPU) filament (ERYONE, Shenzhen, China). TPU was selected to make the ribcage flexible but strong, allowing for repeated deformation without breaking, such as during rib retraction. The pelvis, spine, and model base, as well as 3mm laparoscopic instrument ports, were printed using a rigid polylactic acid (PLA) filament (ERYONE, Shenzhen, China).

**Fig. 1:**
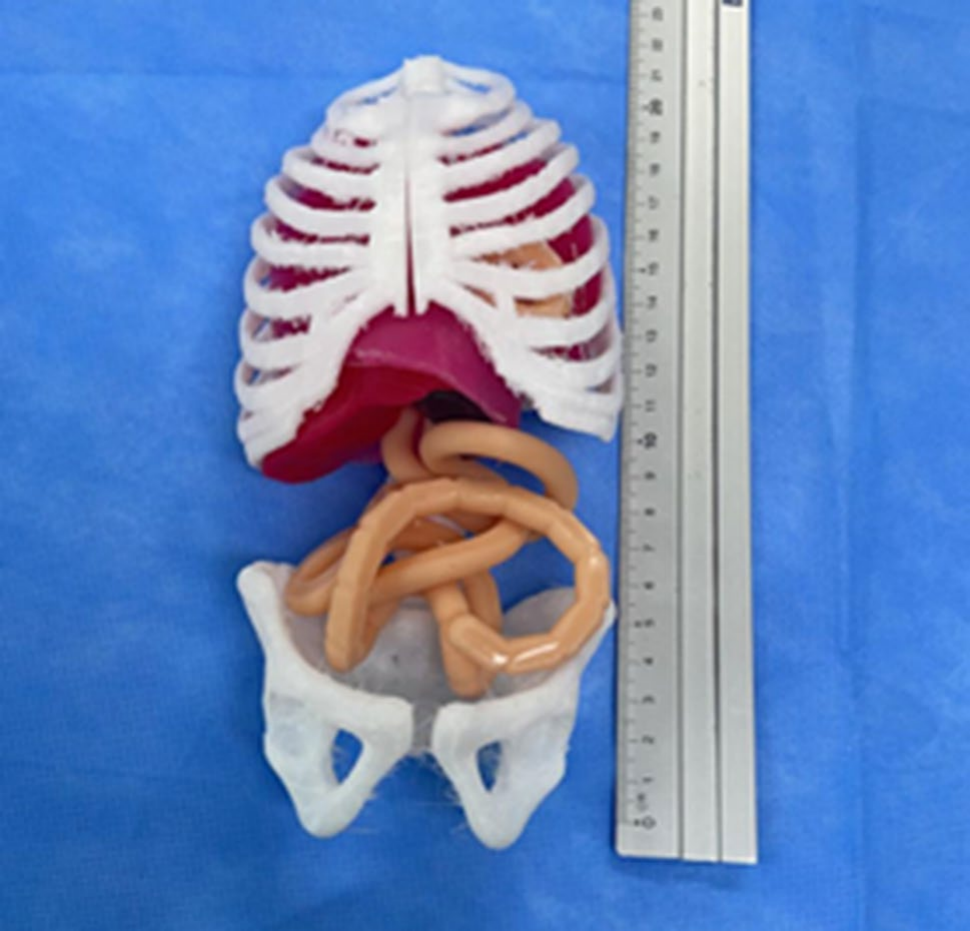
Model without overlying skin showing the 3D-printed components and applied silicone viscera

**Fig. 2:**
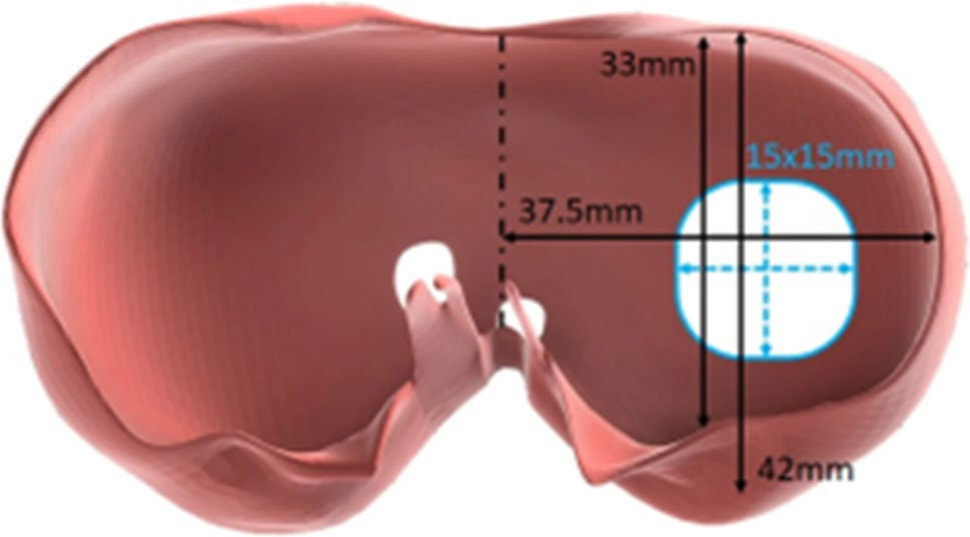
Diaphragm measurements for the repair via laparotomy and thoracoscopy models with defect size (15 mm by 15 mm)

Using measurements derived from the foetal MRI, moulds for silicone casting of soft organs and tissues were 3D-printed, including diaphragm, lung, liver, spleen and bowel. Platinum-catalysed silicone (Smooth-On Inc., Pennsylvania, United States) of varying hardness, and dyed with pigment, was used to maximise realism. The silicone components were applied to the 3D-printed model (Fig. 1). The silicone diaphragm was constructed based on measurements from the patient MRI. These measurements were replicated in the CAD software to create a diaphragm measuring 42 mm by 75 mm (Fig. 2), therefore, each hemi-diaphragm measured 42 mm by 37.5 mm. The entire diaphragm was used in the model of repair via laparotomy and the left hemi-diaphragm was used in the thoracoscopic repair. The defect was measured on the MRI and was added to the silicone diaphragm after curing. This measured 15 mm by 15 mm.

These two models assess several steps in the CDH repair procedure. The model provides anatomical landmarks for planning access. The models simulate: incision or thoracoscopic port placement, navigation of the chest cavity with a camera, identification of the defect, reduction of its content, assessment of the defect size, direct primary closure or patch placement and wound closure. Additionally, the models can be reused with replacement of parts that are sutured or incised.

### Setting and participants

Consultant paediatric surgeons and trainees of all grades were approached at the 68^th^ British Association of Paediatric Surgeons Annual Congress in Birmingham, UK over 3 days in July 2022. Verbal consent was obtained for participation.

### Data collection

Two models were available for participants to measure. Participants were asked to measure the diaphragmatic defect in two-dimensions (2D) along with a 2D measurement of the ipsilateral diaphragm in both models. The instruction cards for the measurements used in the Defect study were provided [6].

**Fig. 3:**
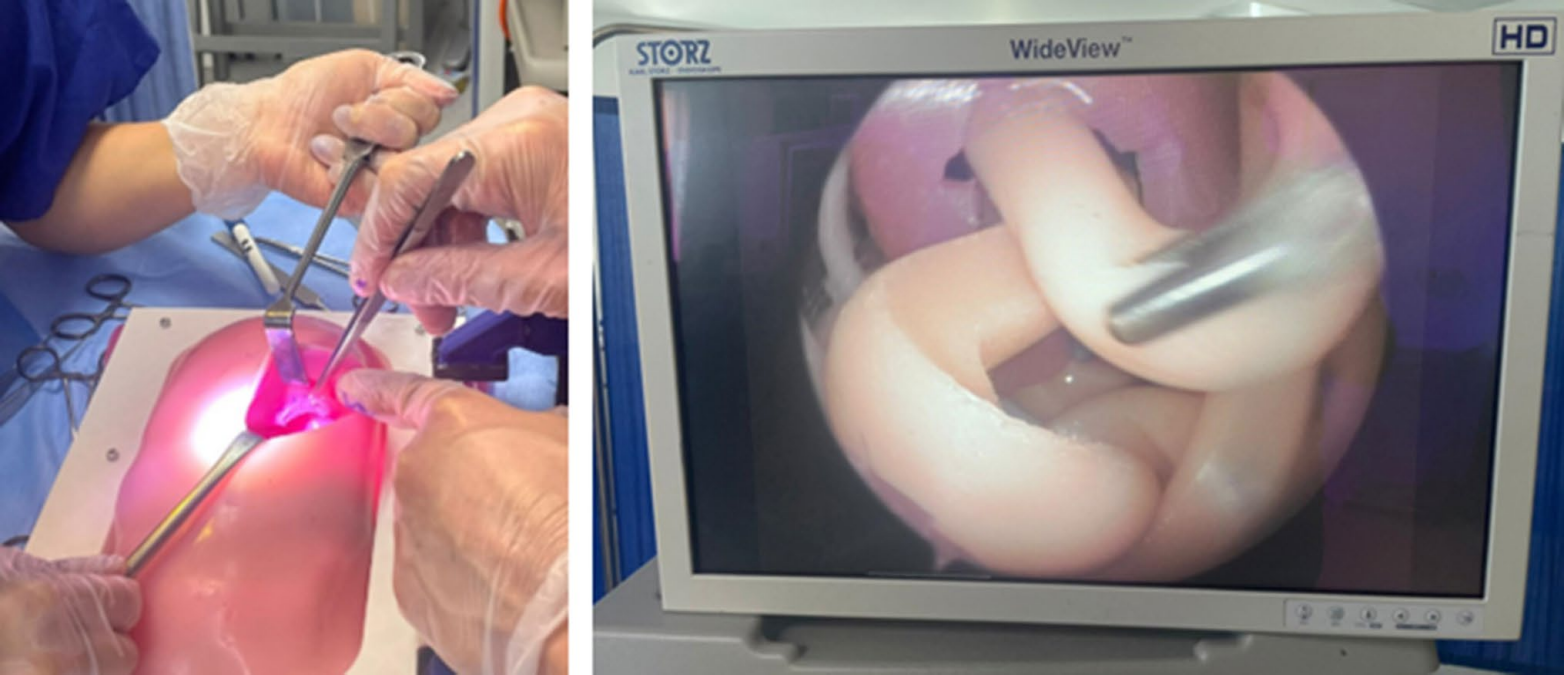
CDH repair model in use via laparotomy (LEFT) where hernial contents have been reduced and the defect closed with sutures. Thoracoscopic view (RIGHT) showing reduction of bowel from diaphragmatic defect

For the thoracosopic model, a 5 mm camera port and two 3 mm axillary ports (Storz) were pre-sited in the left chest wall (Fig. 3). An assistant held the camera whilst 3-mm instruments (thoracoscopic graspers), a braided suture and a measuring tape were made available for the measurement. For the laparotomy repair model, the abdominal wound was pre-sited(Fig. 3). Instruments provided included a measuring tape, two haemostatic clamps and a braided suture. Each participant was invited to measure both the defect and hemithorax in anterior–posterior (AP) and medial–lateral (ML) plane in each model.

The outcome of interest was the accuracy of the diaphragmatic defect measurement in each model in millimetres (mm). Measurements were recorded anonymously alongside grade of seniority and prior operative experience.

### Statistical analysis

Data are presented as median (range) and number/total (%) as appropriate. Mean mms of error per measurement were calculated for both the approach via laparotomy and thoracoscopy per participant. The Mann–Whitney *U* test was used for non-parametric continuous data and linear regression was used to determine relationship between surgeon experience and accuracy of measurement. A *p* value of less than 0.05 was considered as statistically significant. Analysis was undertaken using R (R core team, 2017) and figures were produced using GraphPad Prism 9 (GraphPad Software, La Jolla California USA).

## Results

### Participants

There were 52 participants of whom 20 were consultant paediatric surgeons and 32 were paediatric surgical trainees (3 CT1-2, 16 ST3-5, 11 ST6-8 and 2 not specified). The median number of previous cases undertaken as operating surgeon via laparotomy was 20 (5–370) and 2 (0–20) via a thoracoscopic approach for consultants, whilst it was 2 (0–37) and 0 (0–3), respectively, for surgical trainees. Forty (77%) of participants had contributed to the Defect study.[6].

### Outcome

**Fig. 4:**
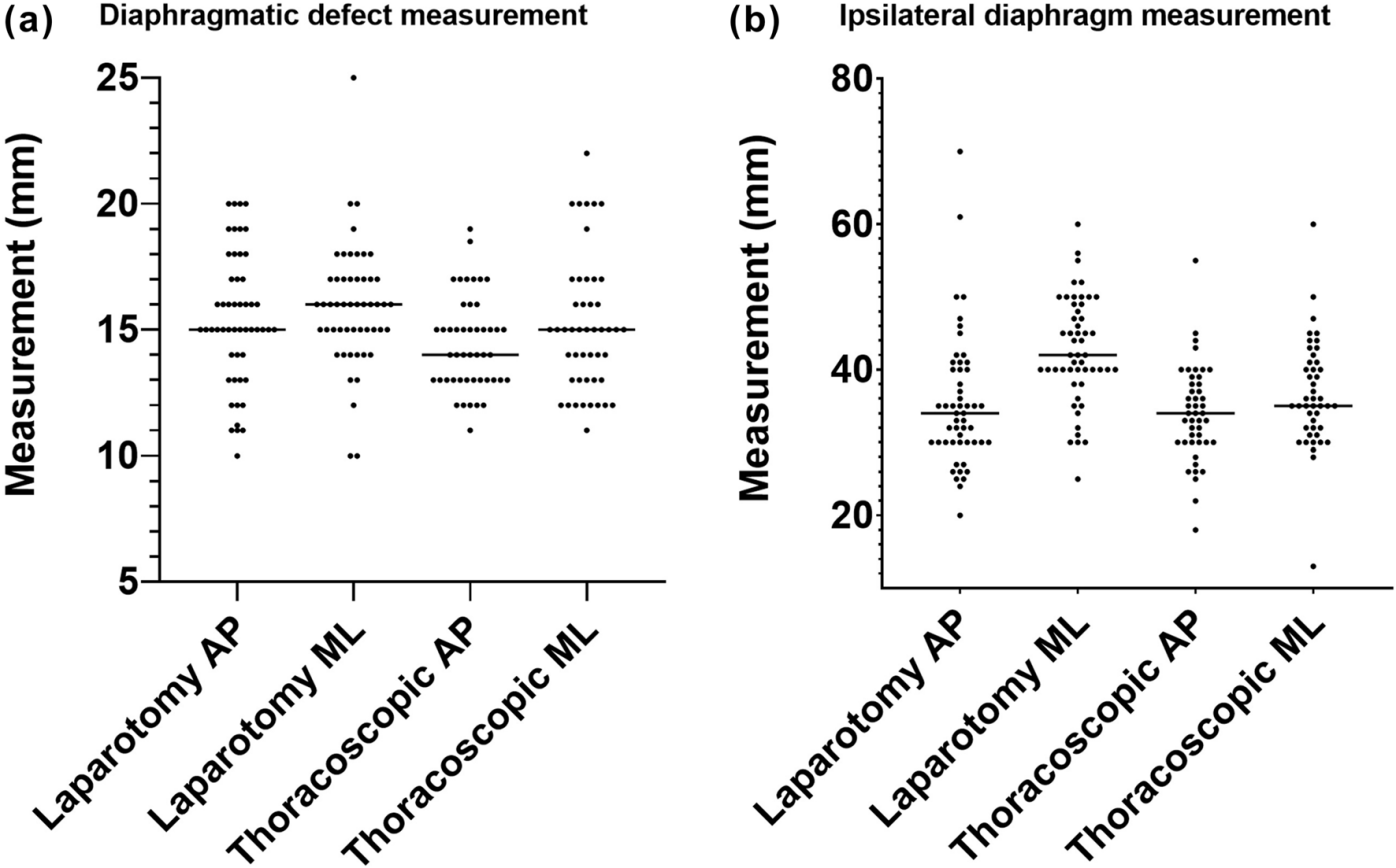
Measurements of **a** diaphragmatic defect and **b** ipsilateral diaphragm via laparotomy and thoracoscopic model. Line is median. *AP* anterior–posterior and *ML* medial–lateral

When undertaken via laparotomy the median diaphragmatic defect AP measurement was 15 (10–20) mm and the median ML measurement was 16 (10–25) mm. When measured thoracoscopically, the median AP was 14 (11–19) mm and ML was 15 (11–22) mm (Fig. 4a).

When undertaken via laparotomy, the median ipsilateral diaphragm AP measurement was 34 (20–70) mm and the median ML measurement was 42 (25–60) mm. The median AP was 34 (18–55) mm and ML was 35 (13–60) mm when measured thoracoscopically (Fig. 4b).

**Fig. 5:**
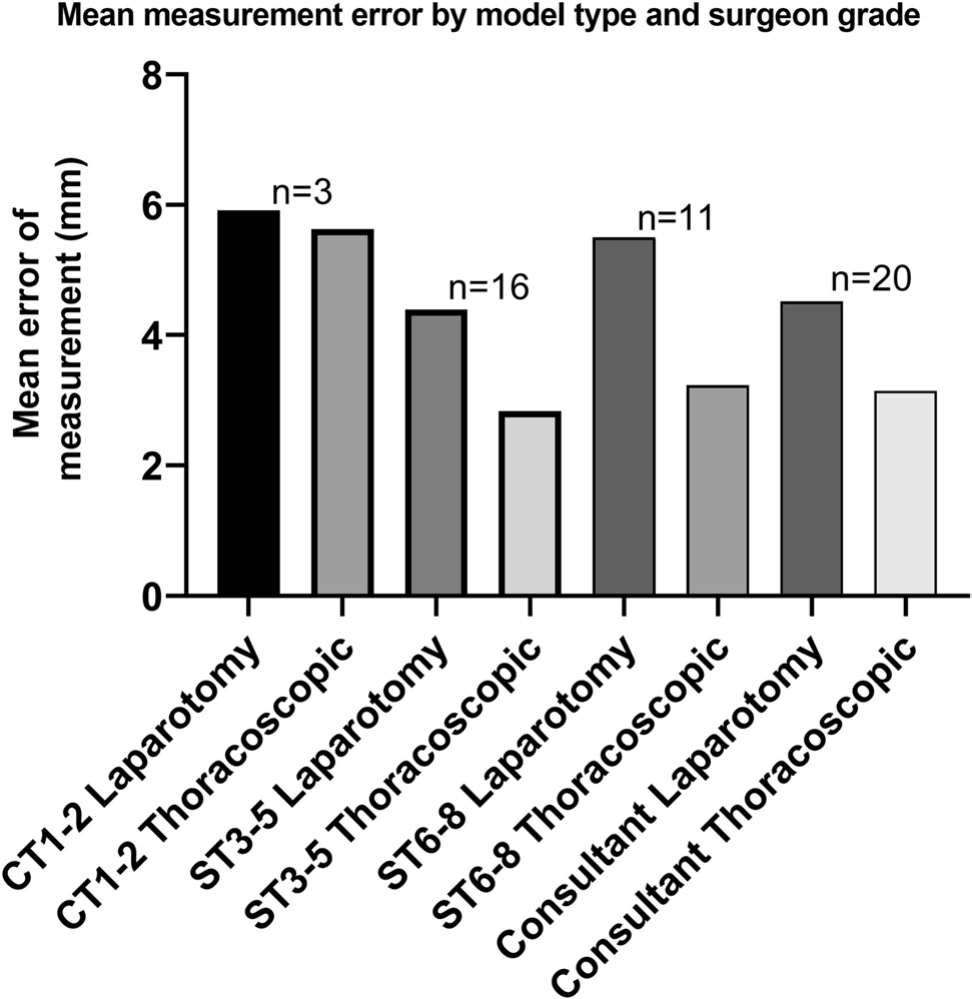
Mean error across all measurements by grade of surgeon and model type. Mean error was similar for all grades of surgeon for model of laparotomy (*p* = 0.59) and thoracoscopic (*p* = 0.42) repair

The mean error per measurement of the defect was 4 (1–17) mm for via laparotomy and 3 (0.5–9.5) mm for the thoracoscopic approach. This measurement of error was similar for consultants compared with trainees for both approach via laparotomy (4.3 vs 3.9 mm, *p* = 0.70) and thoracoscopy (3 vs 3 mm, *p* = 0.79). Additionally, this measurement was similar across all training grades for both approaches (Fig. 5). Error did not correlate with number of procedures undertaken as operating surgeon for either the measurement via laparotomy (*β* = 13.0 [95% CI − 55.9 to 82.0], *p* = 0.71) or thoracoscopic approach (*β* = 1.4 [95% CI − 6.4 to 9.2], *p* = 0.73.

## Discussion

In this study, 3D-printed models for thoracoscopic and open CDH repair have been successfully developed. Interobserver variability has been tested in this simulated model for both thoracoscopic and abdominal measurements of the diaphragmatic defect and ipsilateral hemithorax. This is the first 3D-printed model of CDH to our knowledge which enables simulated surgical repair, whilst showing that intra-operative measurement of the diaphragmatic defect in CDH is repeatable and not dependent on surgeon experience or operative approach.

A major challenge with interpretation of outcome data in CDH is how the size of the defect is classified. Accurate and reproducible descriptions of the diaphragmatic defect will enable more objective comparison of outcomes between centres, surgeons and techniques. The A to D approach proposed by the CDH study facilitates more reproducible reporting but is likely to oversimplify the complexity of defects encountered [9]. With accurate measurement of the defect and hemi-diaphragm, it may be possible to better describe the diaphragmatic defect and how this relates to the remaining diaphragm (i.e. percentage diaphragmatic loss) [6]. This study demonstrates that intra-operative diaphragmatic defect measurement is reproducible and in real practice a prospective national study (unpublished) of CDH in the UK has found that the anteroposterior defect was successfully measured in 87% procedures [6]. Therefore, it is possible to reliably obtain these measurements by surgeons of variable experiences by either open or minimally invasive techniques. This objective measurement of defect size will assist in future comparison of methods of repair techniques on close to identical defects. The liberal patch use to repair small defects could be interrogated to report on comparable recurrence rates and chest wall abnormalities in those repaired primarily. Without accurate classification of defect size, it is difficult to robustly test these hypotheses. Other postnatal outcomes are clearly also important in CDH such as pulmonary function which can be prognosticated using foetal ultrasound (US) or MRI. However, variability exists between methods of lung measurement and it is unclear what the most effective modality of prenatal imaging is for prognostication of pulmonary function [10]. At the time of set-up of the Tracheal Occlusion to Accelerate Lung growth (TOTAL) trial, the lung examination was determined by US measurement of observed/expected lung area to head circumference ratio (LHR), however, ultimately, LHR was assessed also on foetal MRI in the study [11].

Simulation has many various forms and is well established in paediatric surgery, it also features in the latest version of the paediatric surgery curriculum for trainee paediatric surgeons in the UK and Republic of Ireland [12]. It is particularly important for neonatal surgical procedures which are relatively rare but an essential competency for completion of higher surgical training. 3D-printed models have been utilised for other neonatal surgical conditions. A model for laparoscopic repair of duodenal atresia was developed using real patient radiology and validated for surgical training [13]. On validation, it was felt to be valuable as a method for training in this condition. Findings were even more impressive for a model of thoracoscopic oesophageal atresia with trachea-oesophageal fistula repair which is a challenging training operation in vivo [14]. This CDH model adds to the growing number of simulated models.

There are several limitations of this study. It tests interobserver variability but not other concepts of validity such as face or criterion validity. This model is from a 3.5 kg foetus that would be typical of a term baby with a defect likely amenable to primary repair, however, participants were also able to accurately measure the ipsilateral diaphragm so it is likely that a larger defect could also be measured accurately. Other models of varying sizes should be produced and tested in future. The time taken to measure the defect and hemi-diaphragm were not assessed which, along with bleeding risk, is important particularly in anti-coagulated babies requiring extra-corporal membrane oxygenation (ECMO). However, the study recruited a good number of participants who had a wide range of pre-existing experience. The model was durable with repeated use and has the potential to be a very worthwhile, novel training tool.

## Conclusion

This study is the first of its kind to describe development and early evaluation of a model of neonatal CDH amenable to thoracoscopic and open repair. We believe it can lessen the learning curve for a complex neonatal index procedure for trainee surgeons and further work will evaluate the effectiveness of these models for this purpose. Furthermore, we demonstrate that diaphragmatic defect size can be accurately measured in a simulated setting.

## Data Availability

Data available by reasonable request to the corresponding author.

## References

[1] Long AM, Bunch KJ, Knight M, Kurinczuk JJ, Losty PD, Baps C (2018) Early population-based outcomes of infants born with congenital diaphragmatic hernia. Arch Dis Child Fetal Neonatal Ed 103(6):F517–F522 29305406 10.1136/archdischild-2017-313933

[2] Jancelewicz T, Chiang M, Oliveira C, Chiu PP (2013) Late surgical outcomes among congenital diaphragmatic hernia (CDH) patients: why long-term follow-up with surgeons is recommended. J Pediatr Surg 48(5):935–941 23701763 10.1016/j.jpedsurg.2013.02.005

[3] Tsai J, Sulkowski J, Adzick NS, Hedrick HL, Flake AW (2012) Patch repair for congenital diaphragmatic hernia: is it really a problem? J Pediatr Surg 47(4):637–641 22498374 10.1016/j.jpedsurg.2011.11.054

[4] Suply E, Rees C, Cross K, Elagami H, Blackburn S, Giuliani S et al (2020) Patch repair of congenital diaphragmatic hernia is not at risk of poor outcomes. J Pediatr Surg 55(8):1522–1527 31711747 10.1016/j.jpedsurg.2019.10.021

[5] Tsao K, Lally KP (2008) The Congenital Diaphragmatic Hernia Study Group: a voluntary international registry. Semin Pediatr Surg 17(2):90–97 18395658 10.1053/j.sempedsurg.2008.02.004

[6] Eastwood MP, Harwood R, Rhodes H, Bethell GS, Bradnock TJ, Hall NJ (2022) Multi-centre prospective cohort study of diaphragmatic defect phenotype and repair in neonates with congenital diaphragmatic hernia: ‘The Defect Study.’ J Surg Protoc Res Methodol. 10.1093/jsprm/snab009

[7] Putnam LR, Tsao K, Morini F, Lally PA, Miller CC, Lally KP et al (2016) Evaluation of variability in inhaled nitric oxide use and pulmonary hypertension in patients with congenital diaphragmatic hernia. JAMA Pediatr 170(12):1188–1194 27723858 10.1001/jamapediatrics.2016.2023

[8] Neville JJ, Chacon CS, Haghighi-Osgouei R, Houghton N, Bello F, Clarke SA (2022) Development and validation of a novel 3D-printed simulation model for open oesophageal atresia and tracheo-oesophageal fistula repair. Pediatr Surg Int 38(1):133–141 34476537 10.1007/s00383-021-05007-9 PMC8412403

[9] Ackerman KG, Vargas SO, Wilson JA, Jennings RW, Kozakewich HP, Pober BR (2012) Congenital diaphragmatic defects: proposal for a new classification based on observations in 234 patients. Pediatr Dev Pathol 15(4):265–274 22257294 10.2350/11-05-1041-OA.1 PMC3761363

[10] Wilson L, Whitby EH (2023) MRI prediction of fetal lung volumes and the impact on counselling. Clin Radiol 78:955–959 37813756 10.1016/j.crad.2023.09.006

[11] Deprest JA, Nicolaides KH, Benachi A, Gratacos E, Ryan G, Persico N et al (2021) Randomized trial of fetal surgery for severe left diaphragmatic hernia. N Engl J Med 385(2):107–118 34106556 10.1056/NEJMoa2027030 PMC7613453

[12] Nataraja RM, Webb N, Lopez PJ (2018) Simulation in paediatric urology and surgery, part 2: an overview of simulation modalities and their applications. J Pediatr Urol 14(2):125–131 29456118 10.1016/j.jpurol.2017.12.009

[13] Barsness KA, Rooney DM, Davis LM, O’Brien E (2015) Evaluation of three sources of validity evidence for a laparoscopic duodenal atresia repair simulator. J Laparoendosc Adv Surg Tech A 25(3):256–260 25536230 10.1089/lap.2014.0358

[14] Barsness KA, Rooney DM, Davis LM, O’Brien E (2015) Evaluation of three sources of validity evidence for a synthetic thoracoscopic esophageal atresia/tracheoesophageal fistula repair simulator. J Laparoendosc Adv Surg Tech A 25(7):599–604 25314617 10.1089/lap.2014.0370

